# Understanding violence in the context of psychosis: the contribution of phenomenology and its limitations

**DOI:** 10.1192/bja.2024.40

**Published:** 2024-09-25

**Authors:** Howard Ryland

**Affiliations:** Department of Psychiatry, University of Oxford; NIHR Oxford Health Biomedical Research Centre; and Oxford Health NHS Foundation Trust

## Abstract

Navigating the complex relationship between violence and psychosis can frequently be challenging. Psychiatrists may find assessing and managing the risk of violence in this context daunting. In their article ‘The clinical assessment of violence in the context of psychosis: taking a phenomenological stance’, Andreson et al. helpfully summarise the role that psychopathology can play in this process. While careful elucidation of an individual’s experiences may assist in the nuanced formulation of their risk and could offer a specific focus for interventions, the approach has potential shortcomings in certain settings. For some phenomena the link with violence is unclear and it may be constellations of symptoms that are important. Causal pathways are not always linear and there may be important mediators linking psychopathological features to behavioural outcomes. In the resource limited settings in which many contemporary health services operate, a detailed assessment of psychopathology may be hampered by time or other constraints. Alternative, more scalable solutions may therefore be needed in particular scenarios.

The relationship between violence and mental illness is an emotive topic. This is especially true for psychosis, which is poorly understood by the public and commonly linked to negative attitudes, including the perceived dangerousness of people experiencing psychotic disorders. It has long been recognised that people with psychosis have a low absolute risk of perpetrating violence and are indeed at much greater risk of experiencing violence. Despite this individuals with psychosis continue to be widely feared. In acknowledgement of this the authors of ‘The clinical assessment of violence in the context of psychosis: taking a phenomenological stance’ open by stating ‘Although most patients experiencing psychosis are not violent, a diagnosis of a psychotic disorder is associated with an increased likelihood of violence.’ (Anderson et al., 2024)

The robustness of the link between psychosis and violence has recently been challenged by those who argue confounding has not been adequately accounted for in previous study designs (Fusar-Poli et al., 2023). Critics also question the wisdom of publicising such an association, suggesting this would inevitably lead to worse stigmatisation for people with mental illness, especially those from marginalised groups. Others have highlighted that recognition of a link presents an opportunity to intervene, given that treatment appears to reduce the risk of violence (Whiting et al., 2024). Treatment is often readily available for individuals identified as experiencing psychosis, primarily in the form of medications such as antipsychotics and mood stabilisers. Pharmacoepidemiologic studies have shown that people are at lower risk of violence during periods of time when they are treated with these drugs (Fazel et al., 2014). Evidence from the landmark Clinical Antipsychotic Trials of Intervention Effectiveness (CATIE) study corroborates this by demonstrating that poor medication adherence significantly predicts injurious violence (Buchanan et al., 2019). Further research, ideally using prospective methodology could help to elucidate this relationship and shed more light on potential causal pathways, which could in turn support novel therapeutic approaches (De Angelis, 2022).

Violence perpetration by people with severe mental illness has profound consequences for their victims, but also for them as perpetrators, and those around them, such as their family members. It has been estimated to cost £2.5 billon in England and Wales in 2015-16 alone (Senior et al., 2020). Psychiatrists therefore owe a duty to their patients and society to minimise violence by those under their care. The unpredictable nature of risk in any given individual however makes it impossible to be certain whether each patient will act violently or not. It is also incumbent on the psychiatrist to use the least restrictive approach. This tension necessitates a holistic approach which involves curiosity about their patient’s experiences, anchored in other relevant contextual information, leading to thoughtful individual formulation and tailored care planning.

Violence risk has assumed increased importance over recent decades, but can be challenging for psychiatrists to assess and address. This may be partly due to a fear of worsening stigma or of imperilling the therapeutic relationship by asking about violence. It may also be tempered by a belief that violence risk assessment is the domain of the forensic specialist. Lengthy risk assessment tools requiring specialised training may be off putting for the general psychiatrist and have inconclusive evidence for their predictive performance (Ogonah et al., 2023). While some structured risk assessments do include psychopathological features, it is often historical factors, such a previous violence, that are the strongest predictors. Historical or other static demographic factors, have the advantage that they can be relatively straightforward to ascertain and have a reassuring objective stability between clinical raters and across cultures. However, although they may be useful in stratifying patients to guide interventions, these risk factors themselves are by their nature immutable and therefore do not themselves offer targets for treatment at the individual level.

Eliciting phenomenology is one of the core skills of a psychiatrist and is therefore an appealing potential tool for managing people at risk of committing violence. A comprehensive exploration of phenomenology remains essential in developing a nuanced understanding of the patient’s experience to guide treatment, regardless of its relevance in preventing violence. Epidemiological studies have suggested that certain psychopathological features are associated with increased risk, although controversies remain. In one study, persecution, being spied on and conspiracy, were linked to serious violence, but only when mediated by anger (Coid et al., 2013). Such complex relationships necessitate the clinician to be both comprehensive and precise in their mental state examination. The identification of particular combinations of symptoms that predict risk could lead to more robust treatment for those individuals. As well as guiding generic treatments, such as antipsychotic medications, it also raises the possibility of interventions targeted specifically at the relevant psychopathological features. For example, treatments could be focused to reduce anger associated with psychosis. While our understanding of the complex interaction between anger and psychosis is still evolving, new avenues of research could draw on evidence of treatments which may be effective at reducing anger in other populations. Psychological techniques, such as mindfulness (Richard et al., 2022), or novel pharmacological approaches, such as beta blockers (Molero et al., 2023), could be adapted for this purpose and their effectiveness evaluated in people with psychosis.

There are however limitations to risk assessment approaches that rely heavily on the accurate elucidation of complex phenomenology. This requires a lot of time and skill, so may be less useful for screening, for example in primary care or emergency settings, where resources may be more limited and practitioners less experienced in assessing mental illness. Consistent evaluation may also be harder to achieve due to the highly subjective and often fluctuating nature of phenomenology, meaning symptoms may not be reliably elicited in a single, brief assessment. Under such circumstances it may be better to instead employ validated, scalable risk assessment tools based on predictors which can be reliably determined without specialist training.

Violence in people with mental illness is a serious problem and various approaches are needed to manage it effectively. These will necessarily range from highly individualised, phenomenologically informed formations for high risk individuals, to simple, actuarial screening tools at the population level. Such approaches are complementary and should be combined in any comprehensive violence reduction strategy.
